# Current understanding of the human microbiome in glioma

**DOI:** 10.3389/fonc.2022.781741

**Published:** 2022-08-08

**Authors:** Jianhao Liang, Ting Li, Jiajia Zhao, Cheng Wang, Haitao Sun

**Affiliations:** ^1^ Neurosurgery Center, The National Key Clinical Specialty, The Engineering Technology Research Center of Education Ministry of China on Diagnosis and Treatment of Cerebrovascular Disease, Guangdong Provincial Key Laboratory on Brain Function Repair and Regeneration, The Neurosurgery Institute of Guangdong Province, Zhujiang Hospital, Southern Medical University, Guangzhou, China; ^2^ Department of Laboratory Medicine, Clinical Biobank Center, Microbiome Medicine Center, Zhujiang Hospital, Southern Medical University, Guangzhou, China; ^3^ Key Laboratory of Mental Health of the Ministry of Education, Guangdong-Hong Kong-Macao Greater Bay Area Center for Brain Science and Brain-Inspired Intelligence, Southern Medical University, Guangzhou, China

**Keywords:** gut microbiome, intratumoral microbiome, glioma, metabolism, immune microenvironment

## Abstract

There is mounting evidence that the human microbiome is highly associated with a wide variety of central nervous system diseases. However, the link between the human microbiome and glioma is rarely noticed. The exact mechanism of microbiota to affect glioma remains unclear. Recent studies have demonstrated that the microbiome may affect the development, progress, and therapy of gliomas, including the direct impacts of the intratumoral microbiome and its metabolites, and the indirect effects of the gut microbiome and its metabolites. Glioma-related microbiome (gut microbiome and intratumoral microbiome) is associated with both tumor microenvironment and tumor immune microenvironment, which ultimately influence tumorigenesis, progression, and responses to treatment. In this review, we briefly summarize current knowledge regarding the role of the glioma-related microbiome, focusing on its gut microbiome fraction and a brief description of the intratumoral microbiome, and put forward the prospects in which microbiome can be applied in the future and some challenges still need to be solved.

## 1 Introduction

Brain tumor is one of the deadliest cancers, in which glioma is globally recognized as the most common primary brain tumor in the central nervous system (CNS) (1). Gliomas are defined as brain tumors of glial origin (2), which have been divided into 6 different families: (1) Adult-type diffuse gliomas (the majority of primary brain tumors in neuro-oncology practice of adults, e.g., glioblastoma multiforme (GBM), IDH-wildtype); (2) Pediatric-type diffuse low-grade gliomas (expected to have good prognoses); (3) Pediatric-type diffuse high-grade gliomas (expected to behave aggressively); (4) Circumscribed astrocytic gliomas (“circumscribed” referring to their more solid growth pattern, as opposed to the inherently “diffuse” tumors in groups 1, 2, and 3); (5) Glioneuronal and neuronal tumors (a diverse group of tumors, featuring neuronal differentiation); and (6) Ependymomas (now classified by site as well as histological and molecular features) (1). According to the classification of World Health Organization (WHO), glioma can be divided into 1, 2, 3, and 4 grade, and GBM, the most aggressive type of glioma, is classified by the WHO as a grade 4 brain tumor associated with high mortality (1). Currently, the primary treatments for glioma mainly include surgery resection, radiotherapy, pharmacotherapy, etc. (3). Unfortunately, the histological hallmark of GBM include microvascular proliferation, cellular heterogeneity, bilateral invasion, and extensive pseudopalisading necrosis, which are responsible for its invasion, resistance, and recurrence after various therapies (4, 5).In addition, the underlying mechanisms of glioma pathogenesis remain largely unclear.

There are emerging lines of evidence that the human microbiome is highly associated with a wide variety of CNS diseases, including Parkinson’s disease (PD), Alzheimer’s disease (AD), multiple sclerosis (MS), autism spectrum disorder (ASD), stroke, et al. (6–10). However, little attention has been paid to the role of the human microbiome in glioma. The human microbiome may impact tumor biology across multiple tumor types, yet previous studies have not proved the exact mechanisms between the human microbiome and gliomas. Continuous research regarding the microbiome and gliomas is reshaping our understanding of the pathogenesis and treatment of CNS tumors (11, 12).

Increasing evidence has shown that tumor-related microbiome, including the gut microbiome and intratumoral microbiome, may play an indispensable role in pathogenesis and the pathophysiology of gliomas (11–13). The bidirectional interactions between the gut and the brain have been extended to include the gut microbiome, namely “the microbiome-brain-gut axis” (14, 15), where the gut and the resident microbiome have been found to affect the cranial nerve signaling, immune induction and the regulation of the microenvironment of the CNS (16, 17). The crosstalk between the gut microbiome and brain has emerged to show a potential impact on gliomas, whereas more preclinical and clinical research are needed to illustrate involving mechanisms (18, 19).

On the other hand, while the brain was considered to be aseptic, recent studies have found that microbes are also integral components of the brain tissue itself in non-inflammatory and non-traumatic conditions (**Table 1**). Nejman et al. (11) had detected the presence of bacteria in GBM. Besides, studies have shown that tumor-associated microbiome may directly or indirectly regulate the process of disease and responses to treatment of tumors (11, 26). These microbes not only affect metabolic and immune functions of the hosts but also can perceive changes in the microenvironment and respond accordingly (27, 28).

**Table 1:** Summary of recent findings on brain tissue microbiome.

Sample type	Source	Methods	Findings	References
Brain tissue	four different CNS regions of one AD patient and entorhinal/cortex hippocampus samples from an additional eight AD patients	DNA sequencing	*Botrytis cinerea* and *Cryptococcus curvatus* are common to all four CNS regions. Five genera are common to all nine patients: *Alternaria, Botrytis, Candida, Cladosporium*, and *Malassezia*.	(20)
	gray and white matter were studied from 24 AD patients and 18 age-matched controls	DNA sequencing	*E coli K99* and LPS levels are greater in AD compared to control brains. Gram-negative bacterial molecules are associated with AD neuropathology.	(21)
	14 AD patients and 12 matched controls	16S rRNA	AD brains tend to have strikingly large bacterial loads compared to controls	(22)
	Postmortem hippocampal formation specimens from 10 neurological controls,10 AD patients, 22 AD patients and 19 neurological controls from the hippocampus, 12 control and 20 AD cerebellum samples	16S rRNA	Independent of study in both AD and control subjects the most abundant phyla were *Proteobacteria, Firmicutes, Actinobacteria*, and *Bacteroidetes*. Variations in beta diversity between hippocampal and cerebellum samples were observed indicating an impact of brain region on the presence of microbial DNA.	(23)
	10 AD patients and 7 matched controls	16S rRNA	PCR analysis revealed the presence of several bacteria in frozen brain tissue from AD patients. Results show that polymicrobial infections consisting of fungi and bacteria can be revealed in brain tissue from AD patients.	(24)
	711 AD and non-AD control brains	RNA-seq and whole-genome sequencing	HHV-6 demonstrated little specificity to AD brains over controls by either method, while other viruses such as Epstein-Barr virus (EBV) and cytomegalovirus (CMV) were detected at comparable levels. These direct methods of viral detection do not suggest an association between HHV-6 and AD.	(25)
Breast, Lung, Ovary, Pancreas, Melanoma, Bone, and Brain	1526 tumors and their adjacent normal tissues samples across seven cancer types. 643 negative controls	16S rRNA and histological staining methods	Each tumor type has a distinct microbiome composition. The intratumor bacteria are mostly intracellular and are present in both cancer and immune cells.	(11)
Glioma	3 human glioma samples	Tissue clearing, immunofluorescent labeling, optical sectioning microscopy,	the presence of microbiota in human gliomas	(12)

**Figure 1:**
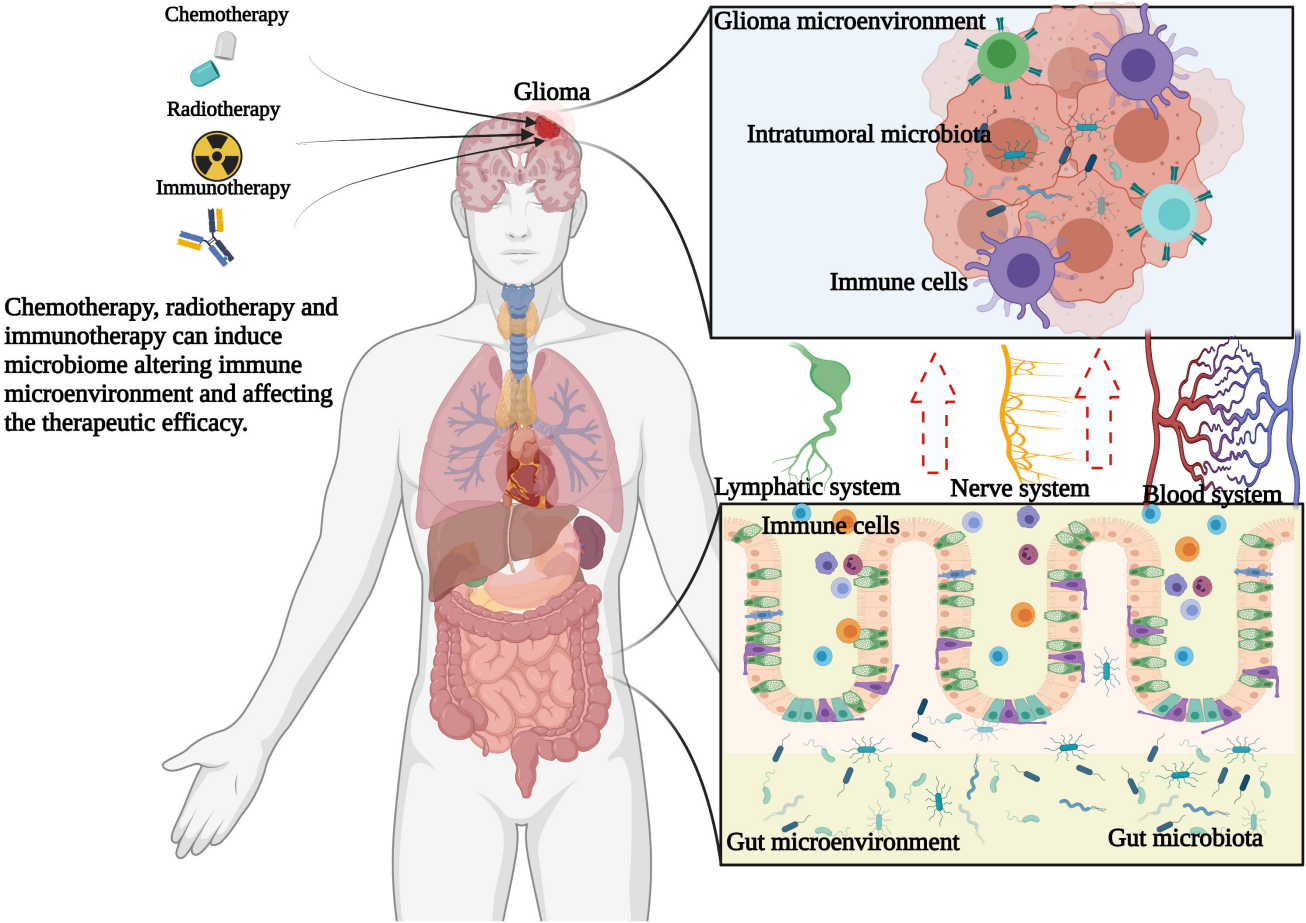
Hypothesis of the relationship among gut microbiome, intratumoral microbiome and glioma. Chemotherapy, radiotherapy and immunotherapy can change the glioma microenvironment and gut microenvironment, thus changing the composition of microbiome, thus shaping the immune microenvironment of glioma and further affecting the therapeutic efficacy.

However, at present, relatively few reports have systematically discussed the role of the gut microbiome and intratumoral microbiome in the initiation, progression, and therapeutic response of gliomas. In this review, we will explain not only the potential relationship between gliomas and the microbiome but also the possible mechanism of the tumor-associated microbiome in the tumorigenesis and development of glioma (**Figure 1**). It is expected to illustrate the potential mechanism of microbial influence on glioma and provide a new direction for the diagnosis and treatment of gliomas.

## 2 The gut microbiome and glioma

The gut microbiome may be involved in the development, progress, and therapy of glioma through metabolic regulation on the epigenetic environments and the immune microenvironment (13, 29, 30). On the one hand, glioma tumorigenesis will change the metabolism of the human body. Glioma cells reprogram metabolism by dysregulating intracellular metabolites, thus glioma cells can proliferate rapidly (31). What’s more, the gut microbiome can regulate the development of glioma by changing the epigenetic landscape of tumor cells (32). On the other hand, the immune homeostasis of the brain requires the gut microbiome to play a role in the function of microglia, T cells, dendritic cells (DCs), and other immune cells (28). In addition, the central nervous system plays a critical role in the microbial composition and function, intestinal barrier, intestinal physiology, and whole-body immune system (33).

### 2.1 Effects of the gut microbiome on glioma by metabolism

Metabolites circulating through blood vessels and lymphatic vessels are one of the important signal molecules produced by the gut microbiome (32), which play an important role in the initiation and development of gliomas (**Table 2**).

**Table 2:** Effects of gut microbiota-metabolites on glioma.

Gut microbiota- metabolites	Mainly involved immune cells or substances	Findings	References
Tryptophan metabolites	T cells/DCs/TAMs antigenpresenting cells (APCs)/astrocytes/microglia	Activating the AHR pathway triggering tumor cell proliferation in astrocytoma, medulloblastoma, and glioblastoma (GBM).	(34)
Arginine metabolites	polyamines and nitric oxide	Polyamine may induce tumor cell proliferation and metastasis by up-regulating the expression of ornithine decarboxylase, spermidine, and spermine acetyltransferase, and Akt1. Nitric oxide can interfere with T cell function by inducing T cell apoptosis. Arginine depletion in GBM can induce excessive autophagy, which will be toxic to tumor cells and may induce apoptosis.	(35–40)
Glutamate metabolites	αKG	Gut microbiome can influence αKG levels through Glu, and changes in αKG affect DNA methylation.	(41)
Glutamine	glutamine	Glioma growth and metabolism are highly dependent on Glutamine and Glutamine starvation therapy has also been shown to reduce the proliferation activity of GBM cells. Changes in the gut microbiome also directly or indirectly alter the content of glutamine in the brain through a variety of pathways, thereby affecting the energy supply of gliomas.	(41–45)
Short-chain fatty acids	acetate, propionate, and butyrate	The imbalance of the gut microbiome, the decrease of the proportion of probiotics, and the lack of abundance of the gut microbiome will lead to the decrease of the concentration of SCFAs in circulation, resulting in the disturbance of morphology and function of microglial cells, resulting in chronic stress status, which affects the development and prognosis of tumors through stress-related pathways. Butyrate affects the immune system by inducing Treg differentiation and regulating inflammation. Acetate and glucose participate in the TCA cycle together, affecting the production of acetyl-CoA in GBM and Acetylation of Rictor by acetyl-CoA actives mTORC2 drives the proliferation and survival of GBM.	(46–49)

#### 2.1.1 Tryptophan

Tryptophan (Trp) has been proved that play a critical role in cell proliferation (50). The metabolism of tryptophan is regulated directly or indirectly by the gut microbiome (51). The metabolites of tryptophan have immune and neuroregulatory functions (52, 53), which may bring new opportunities for the application and transformation of gut microbiome-related research in drug therapy.

The level of free tryptophan *in vivo* is mainly determined by food intake and the activity of three tryptophan metabolic pathways. A very small part of free tryptophan is used in protein synthesis and the production of neurotransmitters (e.g., serotonin) and neuro-regulators (e.g., tryptamine). More than 95% of free tryptophan is metabolized through the tryptophan-canine pathway (53).

The aryl hydrocarbon receptor (AHR) is a ligand-activated transcription factor involved in regulating cell metabolism, proliferation, differentiation, cell death, and cell adhesion (34), which is widely and highly expressed in gliomas, especially in GBM (54). The gut microbiome is critically involved in dietary tryptophan metabolism and catalyzes tryptophan to produce AHR agonists. The latter binds to the AHR of astrocytes and gliomas to trigger related effects, including inducing T cell activation, regulating DC function, and recruiting tumor-associated macrophages (TAMs) through hypoxia-inducible factor 1 (HIF-1) (55). Currently, a study demonstrated a function of the neomorphic enzymatic product of mutant IDH, R-2-hydroxyglutarate (R-2-HG), in regulating amino acid metabolism in immune cells (56). Paracrine R-2-hydroxyglutarate not only further impaired monocyteto-DC differentiation in IDH-mutant glioma but also delays DC maturation and specifically suppresses MHC class I/II-mediated antigen (cross-)presentation and co-stimulation by IL-6, which translates to reduced T cell activating capacities (57). What’s more, R-2-HG is taken up by myeloid cells to enzymatically induce TDO2-dependent activation of the kynurenine pathway and the AHR (56), which excessively degraded tryptophan resulting in an amino acid starvation-like response that triggers the expression of LAT1–CD98, a key transporter for tryptophan in proliferating cells (58), which was previously linked to T cell activation and differentiation (59). At the same time, AHR in DC and TAM can also act on CD8^+^T cells to regulate the growth of glioma (60). What’s more, by consuming endogenous tryptophan, glioma cells activate AHR and to inhibit T cell function, induce T cell apoptosis, promote CD39 expression, and induce the differentiation of T cells mediated by interleukin 10 (IL-10) (61). AHR signaling pathway may modify metabolic pathways associated with amino acid, which inhibits the function of immune cells such as glioma-associated macrophages, T cells, antigen-presenting cells, astrocytes, and microglia, resulting in inhibitory changes in the immune microenvironment during the occurrence and development of gliomas, and promoting glioma invasion and migration (60, 62). The key factors for activation of the AHR pathway include IL4 inducible factor 1 (IL4I1), indoleamine-2, 3-dioxygenase 1/2 (IDO1/2) and Tryptophan 2, 3-dioxygenase (TDO). IL4I1 was positively correlated with AHR activity and negatively correlated with patient survival in both high-grade and low-grade gliomas (63). IDO1/TDO activated the Kynurenine-AHR signaling pathway, which was positively correlated with the pathological grade and Ki67 index of glioma as well as negatively correlated with overall survival (64). In conclusion, AHR is an important factor of the gut microbiome affecting the progression of glioma.

#### 2.1.2 Arginine

Arginine, a semi-essential amino acid in humans, is critical for cell division, healing of wounds, removing ammonia from the body, immune function, and the release of hormones (65, 66). Arginine-derived metabolites, including polyamines and nitric oxide, may affect tumor growth. However, the gut microbiota can absorb dietary arginine to produce polyamines and nitric oxide (67, 68), which are released into the blood circulation system and then transferred to the brain through the blood-brain barrier (BBB). The polyamine may induce tumor cell proliferation and metastasis by up-regulating the expression of ornithine decarboxylase, spermidine, and spermine acetyltransferase, and Akt1 (39). At present, there is still a lot of controversy about the effect of nitric oxide on glioma cells, but its effect may largely depend on the concentration of nitric oxide, exposure time, cell type, and microenvironment (69). In addition, the gut microbiota can produce nitric oxide after reacting with superoxide radicals (70). Nitric oxide can interfere with T cell function by inducing T cell apoptosis (35, 36). Nitric oxide is more likely to cause damage to DNA and mitochondria in tumor cells to enhance the sensitivity of drug-resistant tumor cells to apoptosis during chemotherapy and immunotherapy (71).

In general, arginine starvation may have both beneficial and adverse effects on glioma. On the one hand, the gut microbiome depletes arginine in the tumor microenvironment, which inhibit T cell cycle regulators, thus inhibiting T cell proliferation (72). On the other hand, the consumption of nutritional arginine by the gut microbiome may be beneficial for the eradication of arginine-deficient tumors, which lack the argininosuccinate synthetase converting citrulline to arginine, and therefore can’t meet the high-energy demand in rapid proliferation (37, 38, 73). Arginine depletion in GBM can induce excessive autophagy, which will be toxic to tumor cells and may induce apoptosis (37, 38, 40).

#### 2.1.3 Glutamate

Glutamate(Glu) is the most abundant excitatory neurotransmitter in the brain and plays a crucial role in brain structure and function including learning, memory, emotion, and cognition (74). Alpha-ketoglutarate (αKG), a product of Glu metabolism, is required in DNA demethylation. The gut microbiome can regulate the dynamic balance between αKG and Glu (41), and thus affect DNA methylation. At present, isocitrate dehydrogenase (IDH) gene mutation has been identified as an important biomarker of glioma. IDH1/IDH2 are NADP+-dependent enzymes that catalyze the oxidative decarboxylation of isocitrate to 2-oxoglutarate in cytosol and mitochondria (75). The mutation of the IDH1 and IDH2 protein leads to enzymes with neomorphic enzyme activity that results in the conversion of αKG to the metabolite D‐2‐hydroxyglutarate (D2HG) inhibiting αKG-dependent dioxygenase (76–78). Thus, the mutation of the IDH1 and IDH2 causes abnormal DNA and histone methylation, ultimately leading to widespread hypermethylation of cytosine-phosphate-guanine (CpG) islands (79, 80). The IDH mutations highlight the interaction between metabolism and epigenetics. Thus, the dynamic interaction between the gut microbiome and epigenetic modifications can contribute to regulating glioma growth and development (81).

#### 2.1.4 Glutamine

Glutamine (Gln) is a crucial energy source for glioma cells In addition, Glu and Gln are both involved in energy metabolism and neurotransmission in CNS (82). Starvation therapy has also been shown to reduce the proliferation activity of GBM cells (42–45). However, more than half of the Glnwas synthesized *in situ* in CNS (83), as such the influence of Gln in the peripheral blood on the energy metabolism of glioma seems to be very limited. At present, most of the studies concerning energy metabolism of Gln in gliomas are confined to the CNS. Studies have shown that the Gln level in the peripheral blood circulation of glioma patients is lower than that of normal people (84). Gln may be used to compensate for the excessive energy consumption of glioma cells. The majority of Gln in the gut comes from the diet, and the rest is produced by various types of bacteria in the gut (85). In condition of intestinal lesions such as inflammatory bowel disease or irritable bowel syndrome where the permeability of the intestinal barrier and BBB was increased (86), the glutaminergic receptor in CNS is affected by the microbiome-gut-brain axis and in turn alters the energy metabolism of CNS. Moreover, changes in the gut microbiome can directly or indirectly alter the level of glutamine in the brain (41), ultimately affecting the energy supply of gliomas. Restricting calories and the ketogenic diet, which limit energy metabolism of glioma cells and induce metabolic oxidative stress and apoptosis, may be potential metabolic therapies for glioma (87, 88).

#### 2.1.5 Short-chain fatty acids

Short-chain fatty acids (SCFAs), formed by the fermentation of undigested carbohydrates by gut probiotics such as *Lactobacillus* and *Bifidobacterium*, can influence the glucose and energy metabolism (89, 90). The most common SCFAs included acetate, propionate, and butyrate (91). Studies have demonstrated that SCFAs can enter the circulation through the intestinal mucosa and regulate the maturation process and function of microglia (92). Microglia, known as brain macrophages, are essential for brain development and physiological functions (92). The deficiency of the gut microbiome can lead to defects in the morphology and function of microglia, which can be partly restored by replanting the complex gut microbiome (92). The disturbance of the abundance and composition of the gut microbiome that decreases circulatory SCFAs not only influences microglial maturation and function but also leads to a state of chronic stress, which has a profound effect on the development and prognosis of tumors through stress-related pathways (48).

SCFAs exert both local and systemic effects through comprehensive signal networks, and the main mechanisms involving binding to G-protein coupled receptors and inhibition of histone deacetylase activity (93, 94). These intracellular mechanisms have been found in the gut, gut-related immune tissues, as well as in the nervous system (94, 95). Butyrate affects the immune system by inducing Treg differentiation and regulating inflammation (49). What’s more, SCFAs can traverse the BBB and enter the brain, subsequently modulate the microglia through epigenetic modifications (96). Acetyl-CoA, an optional products of lipids, functions in regulating protein acetylation (97). Acetate is one of the most abundant nutrients in the brain, which can be absorbed by tumor cells, and affect the production of acetyl-CoA in GBM (46). Acetylation of Rictor by acetyl-CoA actives mTORC2, which drives the proliferation and survival of GBM (47).

**Figure 2:**
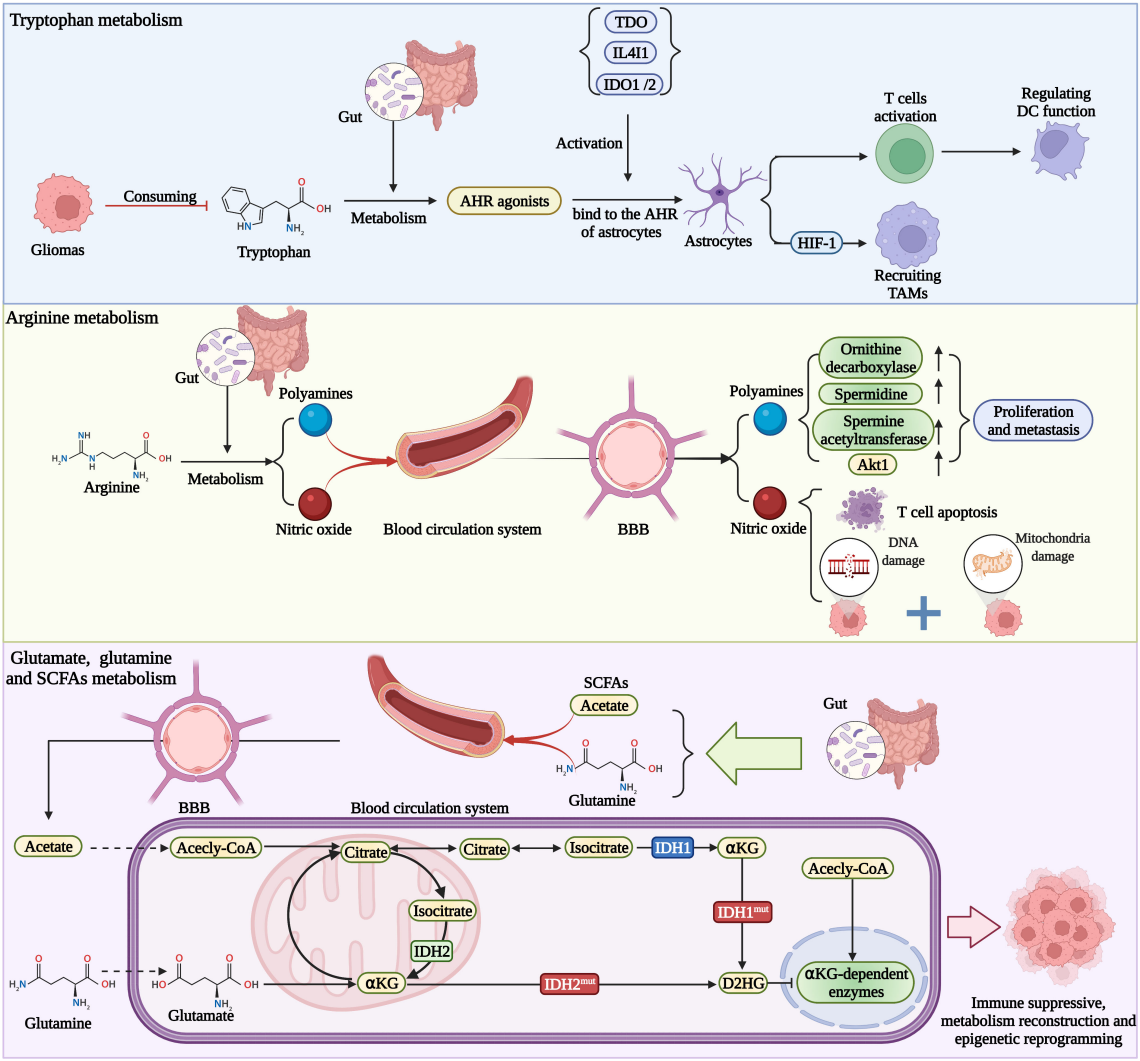
Gut metabolites affect the functions of immune cells and glioma immune microenvironment, which shapes the immune state into the suppressive type. Metabolites also change the epigenetic landscape of glioma cells, then altering the behavior of tumor.

In general, the role of gut microbiota-derived metabolites in the glioma microenvironment can be demonstrated in **Figure 2**. However, it remains to be seen that whether these metabolites produced by the gut microbiome also disrupt the BBB and induce immunosuppression in the brain.

### 2.2 Effects of the gut microbiome on the immune microenvironment of glioma

Although glioma seldom metastasize to other parts of the body, they can be seen as a systemic disease affected by and altering the homeostasis of the body’s immune system (28). The formation of a healthy brain and balanced brain immunity requires the gut microbiome, which plays a role in the function of microglia, T cells, DCs and other immune cells (33).

#### 2.2.1 The transformation of the GBM immune microenvironment

Previously, due to the existence of the BBB and the lack of a classical lymphatic drainage system, the brain has been considered an immune-privileged organ (98). The resultant disruption of BBB during the process of tumorigenesis may permit the entry of peripheral immune cells into the brain microenvironment, such as T cells, macrophages, and B cells (99). Specifically, a major character of the GBM immune microenvironment is the TAMs (100). In the process of gliomagenesis, due to the destruction of the BBB, the resulting bidirectional communication between immune cells and glioma cells creates an immunosuppressive microenvironment that promotes the survival and growth of tumor (101). Then, Glioma-associated microglia and macrophages are recruited to glioma tissues and can be polarized into M2-like cells, which become tumor-supportive and immunosuppressive (102, 103). The increase of M2-like cells in the brain was associated with GBM and negatively correlated with the survival time of glioma patients (104). Besides, previous studies have demonstrated that functional lymphatic vessels link CNS with lymphatic drainage, and that its dysfunction may related with brain cancer (105), further, showing the uniqueness of the brain as an immune-privileged organ (106). The lymphatic outflow of cerebrospinal fluid is reduced in GBM, thus more pro-inflammatory and chemokines can be captured in the glioma microenvironment (107).

#### 2.2.2 Effects of the gut microbiome on glioma tumorigenesis and development by the immune system

Present studies have demonstrated that gut commensal bacteria of newborns can affect the development and function of the immune system (108). Moreover, the gut microbiome plays an indispensable role in the induction, training, regulation, and function of the host immune system (109, 110). In the presence of some gut commensal bacteria, human DCs can induce the differentiation of T helper (Th) cells into Th1 and Th17, promoting the secretion of pro-inflammatory factors (111). Studies suggested that *B. fragilis* can induce the differentiation of IL10-secreting Tregs, which can impair the anticancer immunity of Th1 and are related to the progression and invasiveness of gliomas (112). Moreover, interestingly, the certain gut microbiome may lead to immunosuppression, which may result in severe local immunosuppression in patients with GBM (113, 114).

Recent studies have shown that the gut microbiome can affect the morphology and function of microglia. For example, in the absence of particular gut microbiome, the microglia of germ-free (GF) mice altered in their morphological characteristics and gene expression profiles, and their maturation process were inhibited (85, 115). The increase in the number of immature microglia is considered to functionally impair the immune activation and stress response in GF mice, which is involved in the down-regulation of inflammatory factors and the inhibition of the innate immune signal pathway (49, 115). In addition, the entry of the gut microbiome and their metabolites into the systemic circulation can also affect the morphology and function of CNS microglia (116). As aforementioned, SCFAs, fermented by bacteria, can control the maturation process of microglia, whose lack may lead to the disruption of the structure and immune function of CNS microglia (115, 117).

Whereas the exact mechanism of the gut microbiome affecting human brain microglia remains unclear,. the structure and function of microglia are closely associated with the diversity and specificity of the gut microbiome. The smallest colonies of three kinds of bacteria (i.e., *Bacteroides distasonis, Lactobacillus salivarius, and Clostridium cluster XIV*) can maintain the activation and growth of microglia (115). Recently, a study, for the first time, found that two antibiotics that change the distribution of the gut microbiome can promote the growth of gliomas by inducing early damage of NK cells and phenotypic changes of microglia. In conclusion, the regulation of the gut microbiome can induce alterations of microglia and change the immune microenvironment of glioma.

#### 2.2.3 Effects of the gut microbiome on the therapeutic efficacy of glioma by the immune system

It is reported that the gut microbiome plays a therapeutic role in several types of cancer. Antibiotics may impair the efficacy of chemotherapy and radiation since they upset the balance of the gut microbiome (118). Some gut microbiome can metabolize chemotherapeutic drugs, which leads to drug resistance (119, 120). Currently, temozolomide (TMZ) is a first-choice alkylating agent considered as a gold standard chemotherapeutic drug for newly diagnosed and recurrent GBM (121). Although the new alkylating agent TMZ can improve the outcome of GBM patients and has an impact on the treatment of malignant gliomas, GBM is still an incurable disease. Orally administered TMZ is converted to 5-(3-methyltriazen-l-yl) imidazole-4-carboximide (MTIC) in the blood (122, 123). MTIC is broken down to methyldiazonium cation and 5-aminoimidazole-4-carboxamide (AIC) (122). Subsequently, methyldiazonium cation transfers its methyl group to DNA, RNA, and cellular proteins (123). These methyl groups are transferred to the 6th position oxygen atoms of guanine and O6-methylguanines are formed. Methylation on the O6 position of guanine is a cytotoxic lesion, which stimulates the mismatch of nucleotide bases during DNA replication (124). If O6-methylguanine-DNA methyltransferase (MGMT) mediated repair does not occur, mismatch repair(MMR) proteins identify mispairing in the newly synthesized strand and thymine excision or DNA damage, followed by cell cycle arrest, leading to programmed cell death (125). Besides, MGMT promoter methylation can predict responsiveness to alkylating chemotherapies in glioblastoma. MGMT testing to select patients with glioblastoma for clinical trials is feasible, and withholding TMZ from patients without MGMT promoter methylation is justified in this context.

Clinically, a majority of patients do not respond to TMZ during the course of their treatment and the efficacy of TMZ is limited by antibiotics due to the lack of related immune response (126). Activation of DNA repair pathways is the principal mechanism for this phenomenon that detaches TMZ-induced O-6-methylguanine adducts and restores genomic integrity (125). Consequently, much remains to be clarified; the mechanism of chemoresistance and the roles of related molecules including MGMT, mismatch repair enzymes, DNA excision repair enzymes, PARP, p53, ABC superfamily, and apoptosis-related factors. Not only approaches to increase sensitivity to TMZ but also understanding the cellular biology underlying chemoresistance and the stem cell phenotype will be helpful to prolong the survival time of patients with GBM.

Compared with surgical resection and radiotherapy (RT) alone, the combination of RT and TMZ can improve the 2-year survival rate from 10% to 27% (127). Previous studies have demonstrated that the destruction of the gut microbiome affects the anti-tumor effect of chemotherapy by changing the tumor microenvironment (128, 129), and shown that the gut microbiome can regulate the sensitivity of patients to chemotherapy. Cyclophosphamide is another major alkylating anticancer drug, and its anti-tumor efficacy is also affected by the gut microbiome. What’s more, cyclophosphamide is capable of altering the composition of the gut microbiome in healthy mice (130). However, the changes in microbiome induced by TMZ in the treatment of gliomas are rarely studied.

Studies in the glioma mice model have reported that the abundance of *Akkermansia, Bifidobacterium, and Verrucomicrobia* increased at 7 days after TMZ treatment (126), which the abundance of *Anaerotruncus* increased at 21 days after TMZ treatment (126). These results suggest that the gut microbiome may play a crucial role in anti-tumor response to chemotherapy and immunotherapy.

Studies in mice glioma models have shown that *Akkermansia* is involved in glucose and lipid metabolism, thus improving metabolic disorders (131). *Bifidobacterium* plays an anti-inflammatory and immunomodulatory role by inducing regulatory T cells and regulating the release of inflammatory cytokines (132, 133). Besides, *Bifidobacterium* is also capable of producing folic acid, which is intimately linked to the DNA methylation of MGMT (134). The status of MGMT promoter methylation not only is involved in the inhibition of tumor proliferation but also is related to the anti-tumor effect of TMZ. As a result, it may improve the therapeutic efficacy of TMZ by increasing *Bifidobacterium*, which induced methylation of MGMT promoter by producing folic acid. It was also found that glutathione and lipid metabolism pathways were up-regulated after TMZ treatment, suggesting that there was a connection between TMZ, oxidative stress, and fatty acid levels (126). The increase of *Akkermansia* and *Bifidobacterium* and the decrease of *Anaerotruncus* may be one of the mechanisms of anti-tumor response to TMZ therapy. Further studies are required to confirm the role of the specific gut microbiota in the anti-tumor response of TMZ.

The immunosuppressive microenvironment limits the efficacy of GBM immunotherapy. Balancing the abundance and composition of the gut microbiome can reduce immunosuppression in the GBM tumor microenvironment (32). What’s more, GBM can attract CTLA-4-expressing T cells and PD-L1-expressing T cells, resulting in inhibiting the activation and continuation of the immune response of cytotoxic T cells. PD-1 and CTLA-4 cells can produce a strong synergistic inhibitory effect in T cells (135). Currently, immunotherapy strategies of GBMs include monoclonal antibodies (PD-1/PD-L1) that block suppressor T cell pathways (136). Unexpectedly, the anti-tumor effects of *Bifidobacterium* cooperating with the innate immune system and PD-L1 blocking were observed. These studies suggested that oral administration of *Bifidobacterium* in mice can affect the immune microenvironment of glioma, including induction of DC maturation, stimulation of tumor-specific CD8^+^T cells, recruitment of other immune cells, and activation of type I interferon signal, which hinder tumor growth (137). PD-1 blockade therapy combined with antibiotic therapy can damage the therapeutic effect and reduce overall survival (138). On the other hand, the anti-CTLA-4 antibodies therapy induces intestinal mucosal injury, gut microbiome imbalance, and translocation of specific *Burkholderia* and *Bacteroides fragilis*, which can induce IL-12 activation, DC proliferation, activate fecal specific Th1 cells, and work together to create an ideal immune microenvironment for CTLA-4 antibody to stimulate a protective anti-tumor immune response (139). Therefore, the potential effect of the gut microbiome on the treatment and intervention of glioma should be further studied.

Other immunotherapies, such as chimeric antigen receptor T cell (CAR-T) therapy, oncolytic virotherapy, and tumor vaccine therapy, have also been widely studied in the treatment of GBM (140, 141). The first CAR-T cell therapy in GBM, interleukin-13 receptor α 2(IL13Rα2), is the target (142). With the deepening ofresearch, more and more targets have been excavated. Three Phase I trials of CAR T cells targeting IL13Ralpha2, Her2/CMV, and EGFRvIII in the treatment of recurrent GBM have shown promising results (143, 144). However, as there is a lack of clinical efficacy in the application, CAR-T therapy has not been used in the clinical treatment of GBM. Moreover, oncolytic virotherapy and tumor vaccine for GBM treatment is still in the clinical trial stage. None of these treatments have been proved clinically. Currently, few studies are showing that the gut microbiome has an effect on these treatments. Interestingly, studies have demonstrated that the regulation of the gut microbiome can enhance PD-L1 therapy (145). What’s more, there are common immunological characteristics between immune checkpoint therapy and these immunotherapies. Consequently, the regulation of the gut microbiome may have the potential to improve the efficacy of these immunotherapies (146). We speculate that the gut microbiome can be used to maximize the effectiveness of existing anti-tumor approaches, and could even be used as a biomarker to predict the prognosis and therapy response of glioma patients (147). However, further studies are needed to determine the detailed functions of certain gut microbiome components in the treatment of gliomas.

## 3 The intratumoral microbiome and glioma

The gut microbiome is known to modulate anti-tumor immune responses and can predict the efficacy of treating with immune checkpoint inhibitors in cancer patients (148). Nevertheless, the intratumoral microbiome, which directly interacts with the local tumor microenvironment and tumor immune microenvironment, intuitively, may have an immediate and ultimate effect on the progression and therapeutic effect of cancer (149).

### 3.1 The presence of the intratumoral microbiome in glioma

Traditionally, commensal microbiota of tumor-bearing tissues is considered resident only in tumors direct contact with the outside, such as gastrointestinal cancers. However, evidence have been emerging that broader types of cancer, originating from some “sterile” organs, may harbor microbes.

A recent study provided a comprehensive characterization of the intratumoral microbiota with a large cohort across seven tumor types (11). The group took rigorous and systematic methods, combining histological staining, DNA sequencing, and tissue culture. What’s more, this is the first time that bacteria have been reported in GBM with histological evidence, showed that the intratumoral bacteria are predominantly present in the cytoplasm of both immune cells and tumor cells. Bacterial lipopolysaccharide (LPS) and lipoteichoic acid (LTA) are the main components of the cell wall of gram-negative and gram-positive bacteria, respectively. This study adopted immunostaining of LPS and LTA in human GBM tissue sections and reported that bacterial LPS was present but LTA was not in GBM, whereas gram-positive bacteria (corresponding to LTA) were detected by 16S rDNA sequencing. This suggests that intratumoral bacteria may have altered their envelopes and had defects in their cell walls, especially gram-positive bacteria. Since there were reports describing the processing of bacterial LPS by macrophages as very slow (150), LPS signals may therefore be more easily found in cancer cells and immune cells. Although the localization of bacterial LPS and 16S rRNA were examined separately by immunohistochemistry and RNA fluorescence *in situ* hybridization (FISH), the presence of bacteria inside glioma cells needs to be confirmed by transmission electron microscopy (TEM).

In addition, they also revealed the composition of the intratumoral microbiome in glioma by a multiplexed 16s rDNA sequencing method. They profiled 40 human GBM samples and discovered that Simpson diversity index and the numbers of bacterial species of GBM microbiome ranked third out of the seven tumor types. At the phylum level, the microbiota in GBM tissue was predominated by Proteobacteria and Firmicutes, followed by Actinobacteria and Bacteroidetes. However, they didn’t find characteristic communities of bacteria due to lack of contrast with normal brain tissue (11).

Subsequently, Zhao et al. proposed a three-dimensional (3D) quantitative *in situ* intratumoral microbiota imaging strategy that combines tissue clearing, immunofluorescent labeling, optical sectioning microscopy, and image processing, to visualize bacterial components colonized in gliomas in a contamination-free manner. They also demonstrated the irregular shapes and sparse distribution of bacterial LPS signals within human glioma samples, mostly localized near nuclear membranes or in the intercellular space (12). This study provides a novel and promising method to interrogate the direct interactions between the resident microbial community and the tumor microenvironment, and further, push forward the exploration concerning the presence of microbiota in the brain tumors.

Although there are only a few studies on the existence and roles of intratumoral microbiota in gliomas, we believe that it will become one of the key issues in elucidating the pathogenesis and exploring treatments of glioma, given the emerging blockbuster papers interpreting the important physiological roles of microbiota in tumors such as breast cancer.

### 3.2 The possible origins of the intratumoral microbiome in glioma

Exploring possible origins of intratumoral microbiota is another key point, which will help us find ways to detect their presence, determine the cause and effect with tumors, and explore their physiological roles.

The origins of the microbiome in glioma remain to be clarified. Here are some possible sources of the intratumoral microbiome in the brain. One possibility is that bacteria may already exist in brain tissues before the occurrence of the tumor, which subsequently induce the initiation and migration of gliomas. Alternatively, the gliomas may change the local microenvironment, allowing bacteria to invade the tumor from other places. After tumorigenesis, the destruction of the BBB and cell barriers, coupled with relative immunosuppression, may increase the likelihood of bacteria moving through the circulatory system to the normally sterile sites (151). Furthermore, gut bacteria may enter the brain through the vagus nerve innervating the gut or crossing the BBB (152). Besides, bacteria may gain access into the brain through neuronal retrograde transport *via* the trigeminal nerve, olfactory nerve, and facial nerve connected to the brain (153, 154). The finding that intracellular bacteria reside in tumors in the aforementioned study also raises the possibility that these bacteria are transported into tumor-bearing tissues along with the migration of immune and cancer cells (11). However, all of these speculations have yet to be verified.

### 3.3 The potential effect of the intratumoral microbiota on glioma

Studies began to demonstrate that there is a non-negligible correlation between intratumoral microbiome and tumor pathological characteristics. A potential carcinogenesis mechanism of pancreatic cancer has been proposed in a report that the inflammation induced by the innate immune response to pathogenic bacteria was associated with pancreatic carcinogenesis (155). LPS has been shown to promote the development of pancreatic cancer by blocking the MyD88-dependent pathway while blocking TLR4 and MyD88-independent pathway has a protective effect on pancreatic cancer (156). Similar mechanisms concerning the inflammatory response of the innate immune system may be implicated in other types of cancer. Recently, Fu et al. demonstrated that the depletion of intratumor bacteria significantly reduced lung metastasis without affecting primary tumor growth in a spontaneous murine breast-tumor model (157). During metastasis colonization, the intratumoral bacteria carried by circulating tumor cells enhance their resistance to fluid shear stress by reorganizing actin cytoskeleton, thus promoting the survival of host cells. Their further study demonstrated that intratumoral administration of specific bacterial strains isolated from tumor-resident microbiota promoted metastasis in two mouse tumor models with significantly different levels metastatic potential (157). These findings suggest that intratumoral bacteria are functional and may be involved in tumor tumorigenesis and development, which provides a research direction for the mechanism of intratumoral bacteria in the future. Moreover, intracellular microbiome may be a potential target for early prevention of many kinds of cancer metastasis.

In general, research regarding the role of the intratumoral microbiome in the occurrence and development of tumors is still limited, let alone gliomas. Whether the intratumoral microbiome is involved in tumorigenesis or if it is only a bystander effect caused by the tumor microenvironment, which has not been fully clarified. Although, some researchers consider that the intratumoral microbiome may be inducing tumorigenesis through related mechanisms results in genetic alterations and initiation of the glioma. In general, the potential roles of the microbiome in glioma requires to be further validation.

### 3.4 Intratumoral microbiota as a potential biomarker of glioma

There are three types of links between microbiome and cancer, which allows microbiota to be described as potential biomarkers in cancer, including increase or decrease in numbers of specific organisms, the use of a combination model of several organisms as predictors and evaluating their performance, and finally altering in microbial diversity indexes that give an overall landscape of the microbial community (158). These associations can be extended to study and identify specific intratumoral microbiome as biomarkers of gliomas.

In the first approach, specific microbial species and/or their unique metabolites can be analyzed and selected to serve as biomarkers to predict the progression, efficacy, and recurrence of gliomas (159). Another method is to use the combined model of several microbiota to maximize the area under the Receiver Operating Characteristics (ROC) curve or Area Under Curve (AUC) (160). Finally, alterations in microbial diversity indicators are used as an alternative to changes in the microbiome, which may be associated with certain tumor features, independently of what organisms are present or absent (158).

As the host tissue and intratumoral microbiome can be affected by carcinogenesis, the genetic heterogeneity of the intratumoral microbiome may provide an opportunity for the diagnosis and localization of gliomas (161, 162). For instance, the above-mentioned study by Nejman et al. found that the Proteobacteria to Firmicutes (P/F) ratio appears to vary between tumor types and the predicted functions of bacteria is associated with tumor types and subtypes. These correlations between the profile of intratumoral microbiome and its host tumors, identified by the state-of-the-art DNA sequencing and data analyzing technologies, may well serve the clinical diagnosis (11).

So far, there is no evidence in the literature on intratumoral microbiota as a potential biomarker of glioma. And due to relatively low biomass of the tumor microbiome and the possible contamination and interference in the process of sample collection and sequencing, the diagnostic value of the intratumoral microbiome is undermined. Also, due to a requirement of biopsy samples, the diagnostic value of sequencing and analysis-based methods is limited (163). More research is needed to improve experimental methods and procedures if the intratumoral microbiome is to be used to diagnose and predict tumors

## 4 Challenges and perspectives

**Figure 3:**
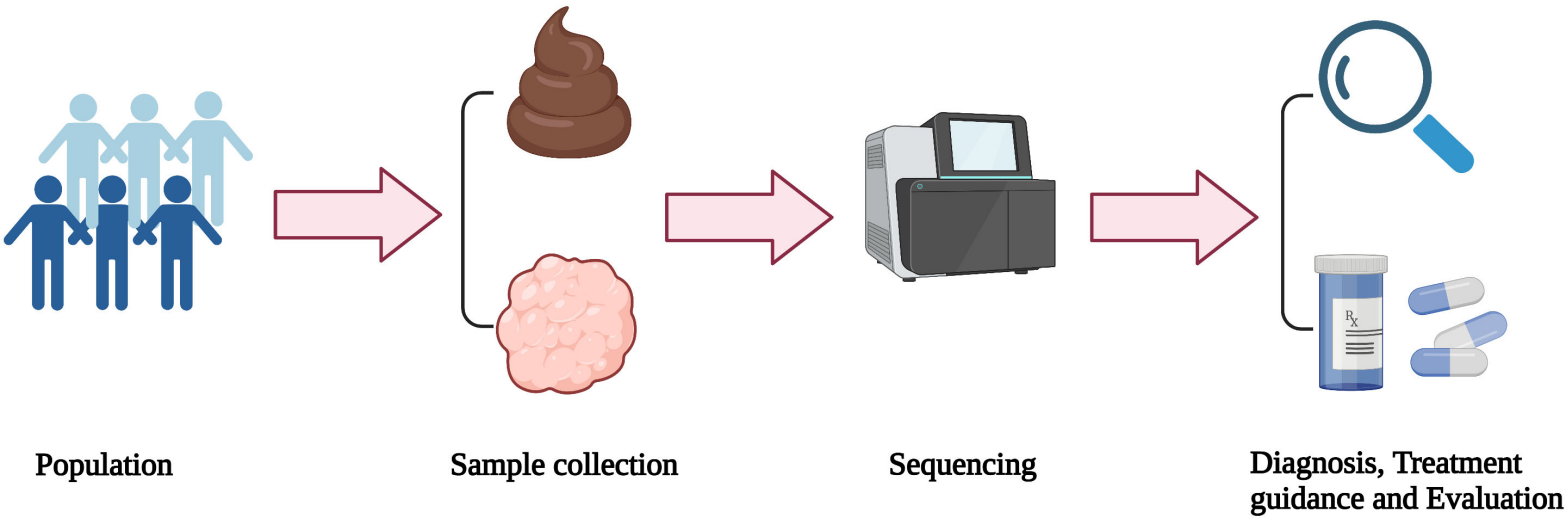
Application and transformation of microbiome. Stool and/or tumor samples from the population are collected and then sequenced. According to the analysis of the sequencing results, glioma patients can be diagnosed and classified, as well as guided personalized treatment.

In the past few decades, microbiome studies have provided a great deal of evidence linking the human microbiome and cancer. The tumor microbiome is claimed to play a role in regulating tumor progression and affect the therapeutic efficacy, which has also been increasingly attracting attention. Therefore, understanding the relationship between microbiome and gliomas is beneficial to elucidate the pathogenesis and corresponding molecular characteristics of gliomas. Moreover, it may eventually be transformed into useful clinical biomarkers for the prevention, diagnosis, and treatment of gliomas (**Figure 3**.). Although the role of the glioma microbiome has not yet been fully determined, mounting evidence and research in these areas are accumulating.

In the one hand, there are still some problems to be discussed between gut microbiome and glioma. Firstly, the relationships between the gut microbiome predicting the efficacy of immunotherapy and race and drug use are not clear. Secondly, the mechanism of the effect of the gut microbiome on the treatment of glioma needs to be further clarified. Thirdly, the timing, dosage, and course of treatment of antibiotics in the process of immunotherapy are still unknown.

In the other hand, there are still many problems to be solved between intratumoral microbiome and glioma. For example, how to avoid potential contamination in the process of collecting tumor tissues, blood, and fecal samples for sequencing remains an outstanding issue (164). In order to enhance the comparability across studies and increase the explanatory power of the research results, it is urgent to establish standardized workflows for tumor microbiome sequencing from sample collection to bioinformatics analysis (165). More preclinical and clinical studies are needed to evaluate not only the composition of the intratumoral microbiome but also the function and multi-omics information of the tumor microbiome. At present, there is still a lack of longitudinal studies to monitor ongoing changes in the microbiome during cancer progression. In addition, changes in external factors may also lead to the alteration of the microbiome composition over time, which may affect the results of microbiome manipulation strategies (166).

Moreover, special attention should be paid to in-depth mechanistic studies to better determine the relationship between microbiome and carcinogenesis. Despite the ability of genomic sequencing technology in profiling the taxonomic diversity of the complex microbiome with low biomass, the sequent loss of spatial information prevents a comprehensive view of the communication between bacteria and cells in the tumor microenvironment. *In situ* bacterial detection techniques, including IHC, FISH, and electron microscopy, etc., have been used to locate bacteria and decipher the host-bacteria communication in tumors. However, these methods are not yet capable of simultaneously detecting multiple markers, and thin tissue slices provide a limited field of view. Emerging advances in multiplexing bacteria probing (167, 168) and three-dimensional visualization of thick tissues (169, 170) are expected to provide an unprecedented insight into the complicated tumor microbiome interactions. Finally, the pioneering spatial multi-omics technology may drive the mapping of the landscape of glioma host-microbiota interactions in the near future (171). The development of organs on a chip (OOCs) provides a brand-new way for experiments (Wu et al., 2020). The OOCs aim to recur the physiological environment and functionality of human organs on a chip by simulating the crucial organotypic cellular architecture and functionality, 3D extracellular matrix, biochemical factors, and biophysical cues. It provides access to the experimental research on the mechanism of microbiome and glioma (Kim et al, 2021).

In addition to the associated reports on gut microbiome and intratumoral bacteria with glioma, some findings revealed that oral microbiota features and gene functions are associated with glioma malignancy and the IDH1 mutation (172). Certainly, animal and cell experiments are further needed to determine the causality of IDH1 mutation on the oral microbiome under glioma status.

To summarize, tumor microbiology is an exciting field to be explored. It is a desire to find new treatment strategies for glioma, including targeted and individualized therapy, to maximize the effect of anti-tumor therapy. More attention is worth paying to explore the value of the glioma-associated microbiome as a potential biomarker of diagnosis, treatment, and prognosis. The establishment of collaborative multidisciplinary networks will be based on enhancing knowledge and optimizing resources. We should spare no effort to overcome the challenges and ensure that we are ready at all times.

## Author contributions

The work presented here was carried out in collaboration among all authors. HS and JL conceived and designed the review; JL wrote the paper. CW and TL helped with literature searching and summarizing. HS and JZ revised the manuscript. All authors read, commented on, and approved this manuscript.

## Funding

This research was supported by Guangdong Basic and Applied Basic Research Foundation (2020A1515010038); Pearl River Science and Technology Nova Program of Guangzhou (201710010047); Presidential Foundation of Zhujiang Hospital of Southern Medical University (No. yzjj2018rc03).

## Acknowledgments

We thank Mr. Jiawen Chen and Miss. Jinyuan Ma for helpful discussions and insightful comments.

## Conflict of interest

Figures in this manuscript were created with BioRender.com.

The remaining authors declare that the research was conducted in the absence of any commercial or financial relationships that could be constructed as a potential conflict of interest.

## Publisher’s note

All claims expressed in this article are solely those of the authors and do not necessarily represent those of their affiliated organizations, or those of the publisher, the editors and the reviewers. Any product that may be evaluated in this article, or claim that may be made by its manufacturer, is not guaranteed or endorsed by the publisher.
